# Elimination patterns of dimetridazole in egg of laying hens and tissues of broiler after oral administration

**DOI:** 10.3389/fvets.2024.1451904

**Published:** 2024-07-23

**Authors:** Kaibin Mo, Chaoqun Wei, Mingyang Bai, Xiangyang Long, Xuezhen Liu, Huanzhong Ding

**Affiliations:** Guangdong Key Laboratory for Veterinary Drug Development and Safety Evaluation, College of Veterinary Medicine, South China Agricultural University, Guangzhou, China

**Keywords:** dimetridazole, laying hens, broiler, residue elimination, HPLC-MS/MS

## Abstract

Dimetridazole (DMZ) is a broad-spectrum anti-anaerobic and antiprotozoal drug extensively used for the control of blackhead disease in poultry (especially turkeys). The presence of DMZ and its metabolites in animal food poses potential risks to human health. In this study, we developed a high-performance liquid chromatography tandem mass spectrometry (HPLC/MS-MS) method for the precise detection of DMZ and its metabolite 2-hydroxymethyl-1-methyl-5-nitroimidazole (HMMNI). Our results demonstrate a strong linear relationship (r^2^ > 0.99) between the concentrations of DMZ and HMMNI in tissues and egg within the range of 1~100 ng/g. The limits of detection (LOD) were determined to be 0.5 ng/g, with corresponding limits of quantification (LOQ) at 1.0 ng/g. Furthermore, average recoveries in tissues and egg fell within the range of 84.90% to 103.01%, with coefficients of variation below 15% for both intra-day and inter-day analyses. To investigate the residue elimination pattern of DMZ and HMMNI, diets containing 500 mg/kg DMZ were fed to healthy SanHuang chicken and Hy-line Gray laying hens for 10 consecutive days. The results indicated that the concentration of HMMNI consistently exceeded that of DMZ during the same period, in both broiler tissues and egg. Sebum showed the slowest elimination of DMZ and HMMNI, becoming undetectable after 168 h of withdrawal. In egg, residues of both substances peaked on the first day after drug withdrawal, followed by slow elimination with half-lives of 0.45 days for DMZ and 0.66 days for HMMNI. Based on these findings, WT1.4 software was used to calculate a withdrawal time of 11 days for broilers and an egg abandonment period of 14 days after withdrawal for laying hens, providing a scientific basis for the safe and rational use of DMZ in poultry farming.

## 1 Introduction

Dimetridazole (DMZ) is a widely used nitroimidazoles drug, exhibiting broad-spectrum anti-anaerobic and antiprotozoal activity, as well as potent inhibition of spirochetes (1, 2), which is commonly used in the treatment of histomonosis (blackhead or infectious enter hepatitis) (3). It is also commonly employed with other antibiotics in clinical settings for the treatment of mixed infections involving both anaerobic and aerobic bacteria (4). DMZ premix have been used as a feed additive due to its efficacy, convenience, and cost-effectiveness (5). However, previous animal studies have found that the potential of genotoxicity could not be judged while carcinogenicity was suggested (6, 7), so DMZ has been banned from use in food-producing animal in some countries (8), or has only been approved for use in the treatment of disease (9, 10).

As early as 1991, G Carignan identified a trace amount of the DMZ N-demethylated metabolite 2-methyl-5-nitroimidazole (2-MNI) in pigs (11). Six years later, Hoogenboom has utilized liver cells to investigate the metabolites of DMZ and determined that 90% of DMZ labeled with 14C was hydroxylated to 1-methyl 2-hydroxymethyl 5-nitroimidazole (HMMNI) (12). A similar study conducted on black tiger shrimp also revealed comparable changes, in which DMZ and its metabolite HMMNI could be detected up to 8 d after administration, despite various cooking methods failing to completely eliminate them (13). In 2007, the Australian Pesticides and Veterinary Medicines Authority (APVMA) established the residual markers of DMZ as DMZ and HMMNI (9).

Due to the limited of data to establish a safe level of residues of DMZ or its metabolites in food, the Joint FAO/WHO Expert Committee on Food Additives (JECFA) currently demand that DMZ should not be detected in all animal foods (14), which imposes higher requirements on the protocols for the use of DMZ in food-producing animal. In order to establish a scientific basis for the safe and rational application of DMZ in poultry farming, our study developed an HPLC–MS/MS method for simultaneous analysis of DMZ and HMMNI concentrations in broiler tissues and egg. Additionally, we investigated the elimination pattern of DMZ in broiler and laying hens using representative SanHuang chicken and Hy-line Gray laying hens as subjects, while also proposing corresponding withdrawal times and egg abandonment periods.

## 2 Materials and methods

### 2.1 Animals and experimental design

In the first experiment, the residues of DMZ and HMMNI in broiler tissues was evaluated using SanHuang chicken sourced from Guangdong Tiannong Food Group Co (Qingyuan, China). Forty-eight healthy SanHuang chickens (120 days old, 1.75 ± 0.15 kg) were randomly allocated into eight treatment groups consisting of six chickens each. The chickens were fed a diet containing 500 mg/kg DMZ for 10 consecutive days, and then at 6, 12, 24, 48, 72, 120, 168, and 240 h after withdrawal, chickens in the relative treatment groups were humanely euthanized, their muscle (pectoral muscle), liver (all), kidney (bilateral, all) and sebum (fat with skin) were collected and stored at −20°C for further analysis.

In the second experiment, the residues of DMZ and HMMNI in egg was evaluated using Hy-line Gray laying hens sourced from Guang Sheng Yuan Agricultural Technology Co (Qingyuan, China). Thirty healthy Hy-line Gray laying hens (300 days old, 1.50 ± 0.20 kg) were individually housed and fed a diet containing 500 mg/kg DMZ for 10 consecutive days. All egg were collected and labeled from day 1 to day 25 of the experiment, with yolks mixed with whites and stored at −20°C for further analysis.

### 2.2 Reagents and chemicals

The DMZ standard was provided by Dr. Ehrenstorfer (Augsburg, Germany) and the HMMNI standard was provided by Anpel Experiment Technology Co., Ltd. (Shanghai, China). The DMZ premix was provided by Wen’s Dahuanong Biotechnology Co., Ltd. (Guangdong, China) and was mixed with the veterinary drugs-free complete formula feed prior to the experiment. All other reagents were of chromatographic purity.

### 2.3 Sample preparation

Accurately weigh 2.0 ± 0.01 g of homogenized broiler tissue and 4.0 ± 0.01 g of egg in a 15 mL centrifuge tube, add 10 mL of ethyl acetate, vortex and mix well, followed by oscillation at 300 rpm for 10 min, then centrifugation at 4°C, 8,000 r/min for 5 min. The extraction process was repeated using the same method and the supernatants were combined, from which an accurate volume of 10 mL was pipetted and evaporated to dryness with N_2_.

The egg extract was redissolved in 1 mL of the initial mobile phase, followed by the addition of 5 mL of n-hexane. The solution was vortexed and mixed thoroughly, then centrifuged at 4°C, 8,000 r/min for 5 min. The lower layer was filtered through a 0.22 μm filter membrane (Anpel, Shanghai, China) and subsequently analyzed using HPLC-MS/MS.

The broiler tissue extract was redissolved in 3 mL of water containing 0.1% formic acid, followed by the addition of 5 mL of n-hexane. The mixture was vortexed, then centrifuged at 4°C and 8,000 r/min for 5 min. The lower layer was subjected to clean-up using an MCX solid phase extraction column (Anpel, Shanghai, China), and the methanol eluent containing 5% ammonia was collected and subsequently dried with N_2_. The resulting residue was redissolved in 1 mL of the initial mobile phase and passed through a 0.22 μm membrane for HPLC-MS/MS analysis.

### 2.4 Liquid chromatography

The compounds were separated on a Phenomenex Luna C18 Column (150 mm × 2 mm, 5 μm) at a temperature of 30°C. The injection volume was set at 5 μL and the flow rate was 0.25 mL/min, controlled by an Agilent 1200 high-performance liquid chromatography (HPLC) system (Agilent, Wilmington, DE, United States). The elution gradient of the HPLC was as shown in Table 1, where the mobile phases consisted of water with 0.1% formic acid (A) and acetonitrile (B).

**Table 1:** Gradient elution procedure.

Time (min)	Flow rate (mL/min)	A (%)	B (%)
0	0.25	90	10
5	0.25	70	30
6	0.25	90	10
8	0.25	90	10

### 2.5 Mass spectrometry

The data were acquired using an API5500 QTrap with ESI source (Applied Biosystems, Foster City, CA, United States) employing the multiple reaction monitoring (MRM) method. The mass spectrometer operated in positive electrospray ionization mode with a turbo ion spray interface maintained at 4,500 V and 500°C. Specific mass spectrometry parameters for each compound can be found in Table 2.

**Table 2:** Detection conditions for DMZ and HMMNI by MS.

Analyte	Retention time (min)	Precursor ion	Fragment ion	DP/V	CE/V
DMZ	4.65	142.2	95.9^*^	74	22
			80.9	80	33
HMMNI	3.85	158.2	140.4^*^	57	26
			54.8	57	18

“*” indicates quantitative ions, DP, Declustering Potential; CE, Collision Energy.

### 2.6 Method validation

The specificity was assessed through comparison of chromatograms obtained from blank broiler tissue and egg, blank broiler tissue and egg spiked with DMZ and HMMNI standard solutions, and broiler tissue and egg samples after administration of DMZ.

The matrix effect was evaluated by calculating the peak area ratios of the DMZ and HMMNI spiked in extracted blank broiler tissue and egg samples with those at the same concentration of 1 and 100 ng/g in pure solution.

Broiler tissue and egg samples with final concentrations of DMZ and HMMNI at 0.5, 1, 5, 10, 50, and 100 ng/g, respectively, were prepared according to the sample preparation and analyzed by HPLC-MS/MS. The signal-to-noise ratio (S/N) ≥ 3 was used as the limit of detection (LOD), and S/N ≥ 10 was used as the limit of quantification (LOQ), the regression equations and correlation coefficients were assessed by linear regression of the peak areas (Y) against the corresponding volume concentrations (X).

The extraction recoveries of DMZ and HMMNI were evaluated by utilizing the final concentrations of 1, 10, and 100 ng/g, respectively, in broiler tissue and egg samples. This was achieved by calculating the ratio of the mean peak area of the analytes in extracted samples to the peak area of the analytes spiked in extracted blank samples at the same concentration. A total of five replicates were conducted for each concentration, with the intra-day and inter-day relative standard deviation (RSD) being assessed.

### 2.7 Sample assay and statistical analysis

The broiler tissue and egg were treated according to the Sample preparation and then assayed by HPLC-MS/MS, and the chromatographic peak areas of DMZ and HMMNI were recorded using Analyst software (version 1.6.2; Applied Biosystems, Inc., Foster City, CA, United States), the regression equations of the standard curves were used to calculate the concentrations of DMZ and HMMNI in the tissues and egg. Some samples with concentrations above the upper LOQ were remeasured after dilution with a blank matrix that had undergone sample preparation. Residual elimination curves were fitted using Excel (Microsoft, United States) software according to the first level elimination kinetic model. Concentrations below the LOD were defined as not-detectable (ND), while concentrations between the LOQ and the LOD were defined as 1/2LOQ. The withdrawal times in broiler and egg abandonment periods in laying hens of DMZ were calculated using WT1.4 software at 95% confidence interval.

## 3 Results

### 3.1 Validation of the bioanalytical method for assaying DMZ and HMMNI concentrations in broiler tissues and egg

**Figure 1:**
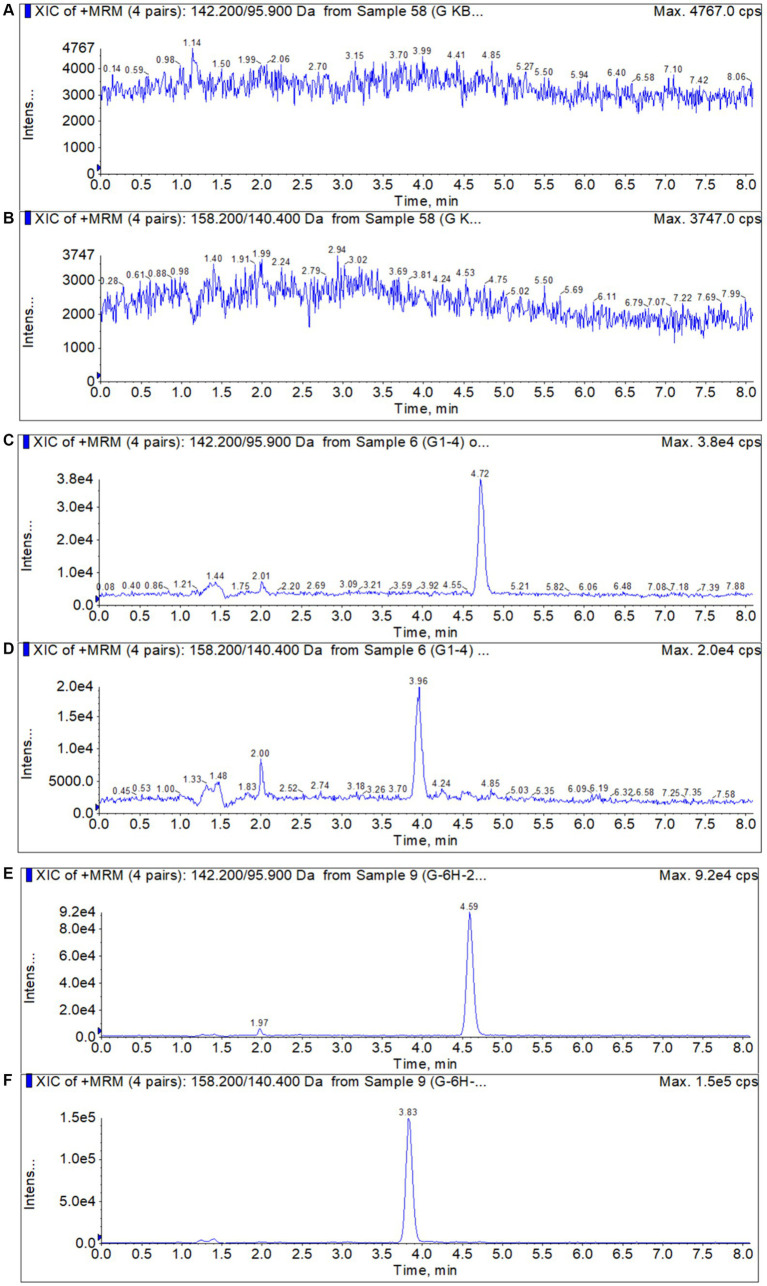
Typical chromatograms of DMZ and HMMNI in liver. **(A,B)** Blank liver sample; **(C,D)** Blank liver sample spiked with 1 ng/g DMZ and HMMNI; **(E,F)** Liver sample after administration of DMZ.

It was shown in Figure 1 that no endogenous compounds interference was identified in the blank samples at the retention times of the DMZ and HMMNI.

The matrix effect was determined by adding DMZ and HMMNI after extracting blank tissues and eggs from six different chickens. As shown in Table 3, the mean matrix effects ranged from 91.85% to 106.68% with RSDs within ±15%, which are within acceptable limits.

**Table 3:** The matrix effects of the method for the assay of DMZ and HMMNI in different tissues (mean ± S.D., *n* = 6).

Tissue	Analyte	Spiked concentration (ng/ g)	Matrix effect (%)	RSD (%)
Muscle	DMZ	1	99.26 ± 9.00	9.07
		100	100.12 ± 6.85	6.84
	HMMNI	1	102.53 ± 6.14	5.99
		100	106.68 ± 3.54	3.32
Liver	DMZ	1	97.54 ± 4.60	4.71
		100	95.73 ± 3.25	3.39
	HMMNI	1	102.98 ± 4.78	4.64
		100	101.88 ± 4.60	4.51
Kidney	DMZ	1	91.85 ± 10.56	11.50
		100	97.32 ± 5.68	5.84
	HMMNI	1	102.12 ± 8.47	8.29
		100	100.81 ± 1.91	1.90
Sebum	DMZ	1	96.96 ± 7.62	7.86
		100	99.91 ± 5.39	5.40
	HMMNI	1	102.43 ± 4.22	4.12
		100	100.25 ± 2.62	2.61
Egg	DMZ	1	96.20 ± 7.57	7.87
		100	99.13 ± 4.49	4.53
	HMMNI	1	99.08 ± 9.81	9.90
		100	99.62 ± 5.92	5.94

In broiler tissues and egg, a curve of DMZ and HMMNI over the range of 1 to 100 ng/g had exhibited a strong linear relationship with the correlation coefficients greater than 0.99. Meanwhile, the LOD of both analytes was 0.5 ng/g, and the LOQ was 1 ng/g for DMZ and HMMNI.

As shown in Table 4, the average recoveries of DMZ and HMMNI in broiler tissues and egg ranged from 84.90% to 103.01%, intra-day and inter-day RSD were less than 10% and 15%, respectively, which indicated that the accuracy and precision of this method were within the acceptable range, as well as meeting the requirements for the analysis of DMZ and HMMNI in broiler tissues and egg.

**Table 4:** The accuracy and precision of the method for the assay of DMZ and HMMNI in different tissues (mean ± S.D., *n* = 5).

Tissue	Analyte	Spiked concentration (ng/g)	Average recovery (%)	Intra-day RSD (%)	Inter-day RSD (%)
Muscle	DMZ	1	88.31 ± 4.92	4.57	5.57
		10	84.90 ± 5.50	6.21	6.48
		100	91.63 ± 3.53	3.89	3.84
	HMMNI	1	99.23 ± 8.49	3.86	8.55
		10	98.84 ± 5.87	5.91	5.94
		100	98.20 ± 2.50	2.55	2.55
Liver	DMZ	1	92.72 ± 12.39	6.32	13.36
		10	96.63 ± 6.11	4.03	6.32
		100	93.10 ± 5.45	3.59	5.86
	HMMNI	1	97.52 ± 6.00	5.76	6.16
		10	103.01 ± 4.56	3.57	4.42
		100	98.32 ± 5.73	3.47	5.83
Kidney	DMZ	1	90.61 ± 7.87	5.71	8.69
		10	95.90 ± 6.44	6.25	6.71
		100	86.46 ± 4.57	5.57	5.29
	HMMNI	1	99.21 ± 8.51	6.43	8.58
		10	98.34 ± 6.95	2.90	7.06
		100	98.54 ± 4.40	4.80	4.47
Sebum	DMZ	1	94.88 ± 5.89	5.79	6.21
		10	100.82 ± 6.81	2.99	6.76
		100	94.58 ± 7.02	5.61	7.42
	HMMNI	1	100.40 ± 11.21	8.16	11.17
		10	102.06 ± 4.36	4.43	4.28
		100	97.95 ± 5.23	4.45	5.34
Egg	DMZ	1	88.01 ± 4.94	3.49	5.61
		10	87.74 ± 4.70	4.90	5.36
		100	92.17 ± 7.54	3.84	8.18
	HMMNI	1	97.67 ± 10.04	4.04	10.28
		10	94.82 ± 3.36	3.72	3.55
		100	93.92 ± 6.24	4.93	6.64

### 3.2 Elimination pattern of DMZ and HMMNI in broiler tissues

As shown in Table 5, the concentration of HMMNI was exceeded that of DMZ in broiler tissues. DMZ and HMMNI were detected in all four tissues at 6 h after the withdrawal, with the highest concentrations of DMZ in sebum and HMMNI in muscle were observed with the Cmax of 272.33 ± 146.63 ng/g and 699.5 ± 275.7 ng/g, respectively.

**Table 5:** Concentrations of DMZ and HMMNI in broiler tissues (mean ± S.D., *n* = 6).

Time (h)	Muscle (ng/g)			Liver (ng/g)			Kidney (ng/g)			Sebum (ng/g)		
	DMZ	HMMNI	Total	DMZ	HMMNI	Total	DMZ	HMMNI	Total	DMZ	HMMNI	Total
6	99.73 ± 26.98	699.5 ± 275.70	799.23 ± 281.1	4.12 ± 3.27	23.42 ± 18.84	27.53 ± 21.51	1.09 ± 0.59	1.76 ± 0.90	2.84 ± 1.37	272.33 ± 146.63	272.60 ± 133.95	544.93 ± 257.61
12	ND	28.14 ± 29.32	28.14 ± 29.32	ND	2.27 ± 2.46	2.27 ± 2.46	ND	0.50 ± 0.00	0.50 ± 0.00	25.16 ± 15.45	74.57 ± 51.73	99.72 ± 53.85
24	ND	1.16 ± 0.11	1.16 ± 0.11	ND	0.50 ± 0.00	0.50 ± 0.00	ND	ND	ND	4.00 ± 1.41	3.97 ± 1.89	7.97 ± 2.96
48	ND	ND	ND	ND	ND	ND	ND	ND	ND	3.51 ± 1.73	3.20 ± 5.65	6.72 ± 7.33
72	ND	ND	ND	ND	ND	ND	ND	ND	ND	3.23 ± 2.51	3.02 ± 2.94	5.04 ± 5.14
120	ND	ND	ND	ND	ND	ND	ND	ND	ND	3.41 ± 2.07	2.1 ± 1.73	4.81 ± 3.29
168	ND	ND	ND	ND	ND	ND	ND	ND	ND	ND	ND	ND
240	ND	ND	ND	ND	ND	ND	ND	ND	ND	ND	ND	ND

ND means not detected.

As shown in Table 6, although the initial concentration of HMMNI was the highest in muscle, it was the most rapidly metabolized that undetectable after 48 h of withdrawal, with an elimination half-life of 2.04 h. In comparison, DMZ and HMMNI residues in sebum were eliminated over a longer period of time, with an elimination half-life of 7.70 and 6.93 h, respectively, and were undetectable after 168 h of withdrawal.

**Table 6:** Elimination parameters of DMZ and HMMNI in broiler tissues.

Tissue	Analyte	Equation of elimination	Elimination constant	Elimination half-life (h)
Muscle	DMZ	——	——	——
	HMMNI	C = 3445.30e^−0.34t^	0.34	2.04
Liver	DMZ	——	——	——
	HMMNI	C = 49.902e^−0.20t^	0.20	3.46
Kidney	DMZ	——	——	——
	HMMNI	C = 6.1952e^−0.21t^	0.21	3.30
Sebum	DMZ	C = 130.94e^−0.00t^	0.09	7.70
	HMMNI	C = 225.90e^−0.10t^	0.10	6.93

### 3.3 Elimination pattern of DMZ and HMMNI in egg

As in broiler tissue, HMMNI concentrations in egg was exceeded that of DMZ as shown in Table 7. The total concentration of DMZ and HMMNI in egg increased rapidly from 96.94 ± 50.09 ng/g to 3864.85 ± 1360.94 ng/g during the first 2 days of administration, and then maintained at a relatively stable value with a certain range of fluctuate from day 3 to day 9. On the day 10 of administration, the concentrations of DMZ and HMMNI showed a relatively large increase and peaked on the day 1 after withdrawal with concentrations of 2347.00 ± 586.23 ng/g and 8944.00 ± 2296.34 ng/g, respectively.

**Table 7:** Concentrations of DMZ and HMMNI in egg (mean ± S.D., *n* = 10).

Period	Time (d)	DMZ (ng/g)	HMMNI (ng/g)	Total (ng/g)
Administration period	1	40.13 ± 18.88	56.81 ± 31.46	96.94 ± 50.09
	2	807.35 ± 330.75	3057.50 ± 1120.68	3864.85 ± 1360.94
	3	733.85 ± 204.58	5655.00 ± 1681.88	6388.85 ± 1838.54
	4	1164.70 ± 245.42	4934.00 ± 1005.82	6098.70 ± 1097.66
	5	1248.60 ± 388.69	4563.00 ± 1285.54	5811.60 ± 1567.31
	6	1162.10 ± 216.75	4821.00 ± 1275.39	5983.10 ± 1277.13
	7	1160.60 ± 367.35	4938.00 ± 890.32	6098.60 ± 1131.59
	8	1340.80 ± 335.05	5391.00 ± 1153.48	6731.8 ± 1413.55
	9	1362.30 ± 329.82	5471.00 ± 1088.63	6833.30 ± 1343.08
	10	2081.00 ± 770.76	8116.00 ± 2680.94	10197.00 ± 3424.04
Withdrawal period	1	2347.00 ± 586.23	8944.00 ± 2296.34	11291.00 ± 2792.87
	2	241.28 ± 86.37	1294.80 ± 398.68	1536.08 ± 470.73
	3	95.59 ± 60.10	420.70 ± 196.04	516.29 ± 254.81
	4	19.95 ± 11.94	89.71 ± 47.92	109.66 ± 59.34
	5	3.97 ± 2.90	23.05 ± 12.42	27.02 ± 15.25
	6	3.01 ± 4.82	13.01 ± 22.65	15.12 ± 26.82
	7	0.69 ± 0.37	4.10 ± 0.88	4.72 ± 1.18
	8	0.69 ± 0.37	3.11 ± 1.81	3.39 ± 2.16
	9	ND	1.88 ± 0.66	1.88 ± 0.66
	10	ND	0.90 ± 0.49	0.90 ± 0.49
	11	ND	1.21 ± 0.61	1.21 ± 0.61
	12	ND	0.84 ± 0.44	0.84 ± 0.44
	13	ND	0.50 ± 0.00	0.50 ± 0.25
	14	ND	ND	ND
	15	ND	ND	ND

ND means not detected.

The results of fitting the residues of DMZ and HMMNI in egg to time are shown in Table 8, the residual elimination of DMZ and HMMNI in egg was slower than in broiler tissues, with elimination half-lives of 0.45 days (10.80 h) for DMZ and 0.66 days (15.84 h) for HMMNI, respectively, which were not detected on the day 9 and day 14 after withdrawal.

**Table 8:** Elimination parameters of DMZ and HMMNI in egg.

Tissue	Analyte	Equation of elimination	Elimination constant	Elimination half-life (d)
Egg	DMZ	C = 8208.10e^−1.53t^	1.53	0.45
	HMMNI	C = 10178.00e^−1.05t^	1.05	0.66

### 3.4 Withdrawal times of DMZ in broiler and laying hens

**Figure 2:**
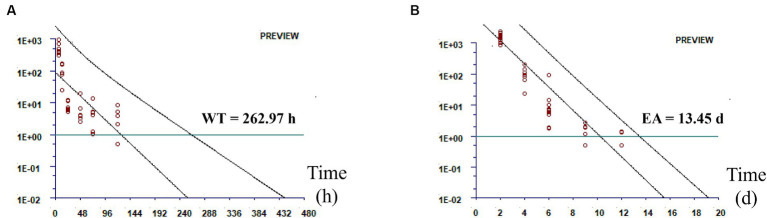
Residual elimination fitting plot. **(A)** Residual elimination of the total amount of DMZ and HMMNI in sebum; **(B)** Residual elimination of the total amount of DMZ and HMMNI in egg.

The total amount of DMZ and HMMNI was used as the residue marker for DMZ, and the temporary maximum residue limit (MRL) was set at 1 ng/g. As shown in Figure 2, the withdrawal time for broilers is 262.97 h and the egg abandonment period for laying hens is 13.45 days after withdrawal.

## 4 Discussion

The main methods for the detection of nitroimidazoles in animal tissues are thin-layer chromatographic (TLC) (15), gas chromatography (GC) (16), high performance liquid chromatography (HPLC) (17) and liquid chromatography tandem mass spectrometry (LC-MS/MS) (13). The HPLC-MS/MS method has been widely used currently because of its high sensitivity, good separation, and conservation (18–20). DMZ and HMMNI are weakly basic compounds which are insoluble in water but soluble in organic solvents such as acetonitrile and ethyl acetate (11, 21). A similar study found that the addition of an appropriate amount of formic acid to the mobile phase improves their ionization efficiency in the positive mode (20), so we choose water with 0.1% formic acid-acetonitrile was used as the mobile phase in this study.

Animal tissues contain more complex components such as sugars, fats, proteins, and vitamins, which can interfere with the detection of trace drugs (22). Therefore, tissue samples should be subjected to extraction and purification procedures before detection. Ethyl acetate and n-hexane are good extractants and purifiers that help to reduce matrix interference with ionization, which is commonly used for extraction of nitroimidazoles (18, 19). In this study, ethyl acetate was used to extract DMZ and HMMNI from broiler tissues and egg firstly, followed by fat removal with n-hexane. The tissue extracts were then cleaned up with MCX solid phase extraction cartridge, and the addition of a certain proportion of ammonia in the final elution procedure, which has been reported to be beneficial to reverse the ionic state of DMZ and HMMNI to improve the extraction recovery (23).

In broilers, DMZ and HMMNI were found to be rapidly and widely distributed in tissues after administration, with HMMNI having the highest initial concentration in muscle but the fastest elimination, with an elimination half-life of 2.04 h, which is similar to the finding of Posyniak et al. (24). However, DMZ and HMMNI had the longest residual time in sebum, with an elimination half-life of 7.70 and 6.93 h, respectively, suggesting that sebum of animals is the main accumulation site of DMZ and its metabolites, which may be related to the better lipid solubility of the compounds themselves (25).

The yolk is formed mainly from lipoproteins synthesized by the liver and transported through the circulatory system to develop in the follicles of the ovary, whereas the albumen consists of water-soluble proteins secreted at the dilated site of the fallopian tube, where the yolk takes the longest time to be formed, approximately 10 days (26, 27). In the present study, it was found that both DMZ and HMMNI accumulated in egg to varying degrees during drug administration, with their concentrations peaking on the first day and becoming undetectable until the 14 days after withdrawal, suggesting that the distribution of drugs between egg yolks and whites is related to the process of egg formation, in addition to the physicochemical properties (e.g., molecular weights, pKa values, and the ability to bind to plasma proteins) of the drugs (28, 29).

Due to the inability to establish an Acceptable Daily Intake (ADI) for DMZ, many countries have not established MRLs for DMZ and its metabolites (10, 30). The shape and parameters of the residue distribution, LOQ and number of trials all affected the accuracy and precision of the estimated MRL (31). It is also feasible to set the LOQ to a temporary MRL when no MRL has been set for the drug for the time being (32, 33). Therefore, we set 1 ng/g as the temporary MRL for the total amount of DMZ and HMMNI. In addition, our results showed that DMZ and HMMNI were rapidly eliminated from muscle, liver and kidney of broilers with no detectable after 48 h of withdrawal, whereas the residual time in sebum was up to 120 h, so we chose sebum as the residual marker tissue of broiler for the withdrawal time analysis of DMZ. Under the conditions of this experiment, we used WT1.4 software to obtain the withdrawal time for broilers is 262.97 h (10.96 days) and the egg abandonment period for laying hens is 13.45 days after withdrawal. For practical purposes, we recommend a withdrawal time for broilers is 11 days and the egg abandonment period for laying hens is 14 days after withdrawal.

## 5 Conclusion

In this study, an HPLC-MS/MS method for the simultaneous assay of DMZ and HMMNI concentrations in broiler tissues and egg was developed for investigating their residue elimination patterns. Residues of HMMNI were consistently higher than those of DMZ in both broiler tissues and egg. Sebum was the accumulating tissue for DMZ and HMMNI and had the slowest elimination rate. In egg, residues of DMZ and HMMNI reached a peak on the first day after withdrawal, followed by slow elimination with elimination half-lives of 0.45 and 0.66 days. Under the conditions of this study, we recommend a withdrawal time of 11 days in broilers and 14 days after withdrawal for egg abandonment period in laying hens.

## Data availability statement

The original contributions presented in the study are included in the article/supplementary material, further inquiries can be directed to the corresponding author.

## Ethics statement

The animal study was approved by Animal Ethics Committee of the Experimental Animal Center of South China Agricultural University. The study was conducted in accordance with the local legislation and institutional requirements.

## Author contributions

KM: Methodology, Writing – original draft, Writing – review & editing. CW: Data curation, Validation, Writing – review & editing. MB: Methodology, Writing – review & editing, Investigation. XLo: Software, Writing – review & editing. XLi: Visualization, Writing – review & editing. HD: Funding acquisition, Resources, Supervision, Writing – review & editing, Conceptualization.
